# Reef Fishes of Saba Bank, Netherlands Antilles: Assemblage Structure across a Gradient of Habitat Types

**DOI:** 10.1371/journal.pone.0009207

**Published:** 2010-05-21

**Authors:** Wes Toller, Adolphe O. Debrot, Mark J. A. Vermeij, Paul C. Hoetjes

**Affiliations:** 1 Saba Conservation Foundation, Saba, Netherlands Antilles; 2 Caribbean Research and Management of Biodiversity Foundation, Willemstad, Curaçao, Netherlands Antilles; 3 Department of Environment and Nature, Ministry of Public Health and Social Development, Willemstad, Curaçao, Netherlands Antilles; University of California San Diego, United States of America

## Abstract

Saba Bank is a 2,200 km^2^ submerged carbonate platform in the northeastern Caribbean Sea off Saba Island, Netherlands Antilles. The presence of reef-like geomorphic features and significant shelf edge coral development on Saba Bank have led to the conclusion that it is an actively growing, though wholly submerged, coral reef atoll. However, little information exists on the composition of benthic communities or associated reef fish assemblages of Saba Bank. We selected a 40 km^2^ area of the bank for an exploratory study. Habitat and reef fish assemblages were investigated in five shallow-water benthic habitat types that form a gradient from Saba Bank shelf edge to lagoon. Significant coral cover was restricted to fore reef habitat (average cover 11.5%) and outer reef flat habitat (2.4%) and declined to near zero in habitats of the central lagoon zone. Macroalgae dominated benthic cover in all habitats (average cover: 32.5 – 48.1%) but dominant algal genera differed among habitats. A total of 97 fish species were recorded. The composition of Saba Bank fish assemblages differed among habitat types. Highest fish density and diversity occurred in the outer reef flat, fore reef and inner reef flat habitats. Biomass estimates for commercially valued species in the reef zone (fore reef and reef flat habitats) ranged between 52 and 83 g/m^2^. The composition of Saba Bank fish assemblages reflects the absence of important nursery habitats, as well as the effects of past fishing. The relatively high abundance of large predatory fish (i.e. groupers and sharks), which is generally considered an indicator of good ecosystem health for tropical reef systems, shows that an intact trophic network is still present on Saba Bank.

## Introduction

Saba Bank, located offshore from Saba Island, Netherlands Antilles, is a large (∼2,200 km^2^), isolated and completely submerged Caribbean atoll [1]. The bank consists of a flat-topped carbonate platform extending to minimum depths of 12 to 50 m below sea level, and no emergent reefs. The geological origin of Saba Bank has been debated [2] but it was ‘undoubtedly’ volcanic [3]. The bank rises steeply from the surrounding sea-floor with extensive coral reef formation at its edges [1], [4]. These observations have led previous authors to conclude that Saba Bank is a submerged coral reef atoll [1], [3].

**Figure 1:**
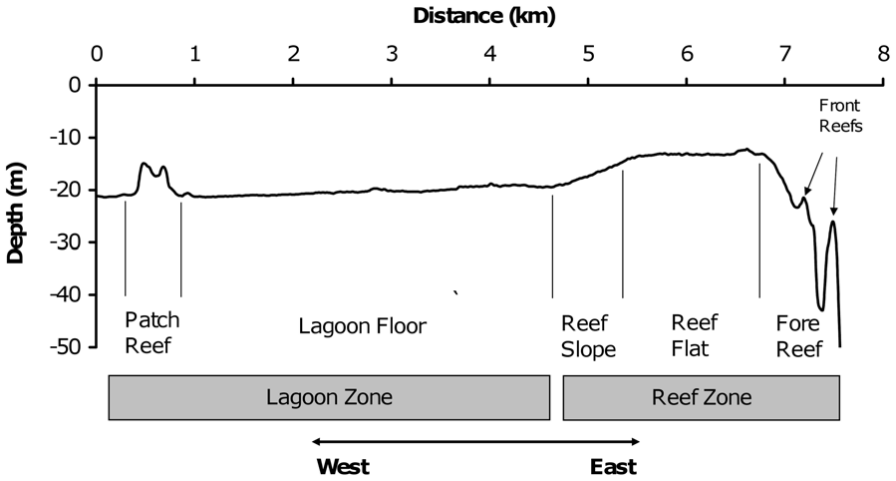
Depth profile across Overall Bank study area, Saba Bank. A depth profile of the study area (∼8 km in length) was generated in ArcGIS using bathymetric data from the Hydrographic Service of the Royal Netherlands Navy. Names of reef structures and reef zones are from Van der Land [3].

Formation of coral reef atolls is relatively rare in the Atlantic [5]. Studies of atolls in the southeastern Caribbean suggest that all Atlantic atolls share a common pattern of geomorphology and reef zonation [6]. The geomorphic features found on Saba Bank were first described by Van der Land [3] who considered the bank to be an actively growing – though wholly submerged - coral reef atoll. At the broadest spatial scale, Van der Land separated the shallow platform area of Saba Bank into a peripheral reef zone which surrounds a large central lagoon zone. At a finer spatial scale, he observed that discrete reef structures occur in a predictable sequence from reef zone to lagoon as follows: seaward slope, fore reef (with one or more “front reefs”[3]), reef flat, reef slope, and lagoon floor with isolated patch reefs (**Figure 1**). This sequence of reef features creates a spatial gradient in habitat type that extends from the rim of Saba Bank to the bank's center.

In tropical marine ecosystems, habitat diversity partially underlies the diversity of organisms as local species richness increases with an increasing number of different critical habitat types [7]. For example, Bellwood and Hughes [8] demonstrated that much variation in biodiversity across Indo-Pacific coral reefs can be described simply by the amount of habitable area in the region (i.e. from island to archipelago scale). The diversity and abundance of coral reef species has been shown to increase with increased refuge availability [9], [10], proximity to nursery habitats [11], [12], or settlement habitats [13]. Successful colonization of distant islands has also been linked to the length of species' pelagic larval duration (PLD) [14]. Clearly, both dispersal dynamics and habitat availability directly affect the number of species found at any particular site [15].

Despite the paucity of data on biogeographical patterns in marine species diversity across the Caribbean, Saba Bank provides a natural setting that is unique in the Caribbean region. The absence of mangroves and sea grasses that are found on many Caribbean islands will likely result in the absence of fish species that depend on such habitats as “nursery areas” [11]. Because coral community development is mainly restricted to the bank's outer rim, fish assemblages likely differ from those occurring on the inner bank lagoon (**Figure 1**) that is characterized by horizontal limestone pavement on which mainly macroalgae and gorgonians are found. Coral communities are structurally more complex than these algal and gorgonian communities and increased structural complexity generally correlates with higher local fish biomass and species richness [16]. On the other hand, Saba Bank as a whole represents a large area which normally results in higher local richness of fish species [15]. The platform area of Saba Bank (i.e. <50 m depth) is similar in size to Caribbean Islands such as Cayman Islands, Grenada and the US Virgin Islands where known fish species numbers are 328, 321 and 381, respectively [15], compared to 270 fish species recorded from Saba Bank [17].

Despite the relatively isolated position of Saba Bank, it has not escaped the effects of anthropogenic disturbance such as fishing for benthic invertebrates (mainly spiny lobster, *Panulirus argus*) and locally abundant fish species (Lutjanidae, Haemulidae, Serranidae and Balistidae) and anchoring and tank cleaning by oil tankers [1], [18]–[20].

Because Saba Bank is difficult to access due to its offshore location, not much is known about the species that are found there. This study aims to determine whether the aforementioned habitat types harbor different fish assemblages in terms of species richness and biomass. The presence of each habitat type was determined using bathymetric maps and remote sensing that led to delineation of five shallow-water benthic habitat types along a gradient from Saba Bank shelf edge to lagoon. Within each habitat type, we conducted underwater visual surveys to examine habitat characteristics and quantify the structure of reef fish assemblages.

## Materials and Methods

### Ethics Statement

All Saba Bank projects have collecting permits through CITES (where necessary) and the Saba Conservation Foundation (where CITES is not required).

### Study Area

**Figure 2:**
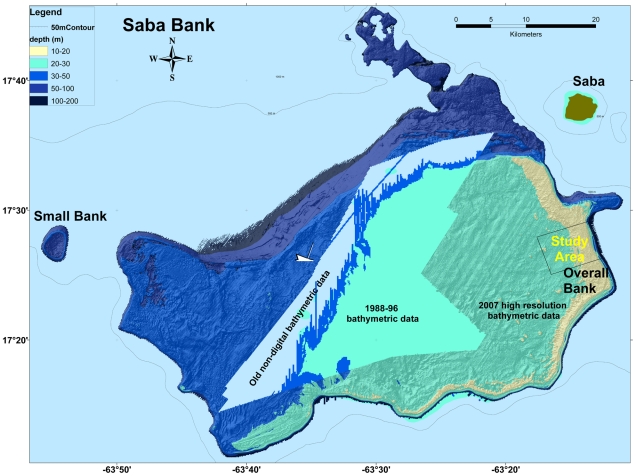
Bathymetric map of Saba Bank. The Saba Bank map was assembled in ArcGIS using available datasets for bathymetry of Saba Bank. Sampling was conducted on a region of Saba Bank named Overall Bank as shown (rectangle at right).

Our study area was situated in a central part of eastern Saba Bank known as Overall Bank, located ∼16 km offshore from Saba Island (**Figure 2**). We selected this study area as a representative section of the ‘Southeastern Reef’ [1], [3] – Saba Bank's largest reef system. The study area was a rectangle 7.3 km long by 5.5 km wide totaling 40.2 km^2^ that extended from the reef edge into the lagoon zone (**Figure 2**). Most of the geomorphological reef features known from Saba Bank (see below) are represented within our study area. The sequence of reef structures with different habitat types is oriented perpendicular to the predominant winds and currents coming from the East. See other papers in this volume for further details on Saba Bank.

### Habitat Types

**Figure 3:**
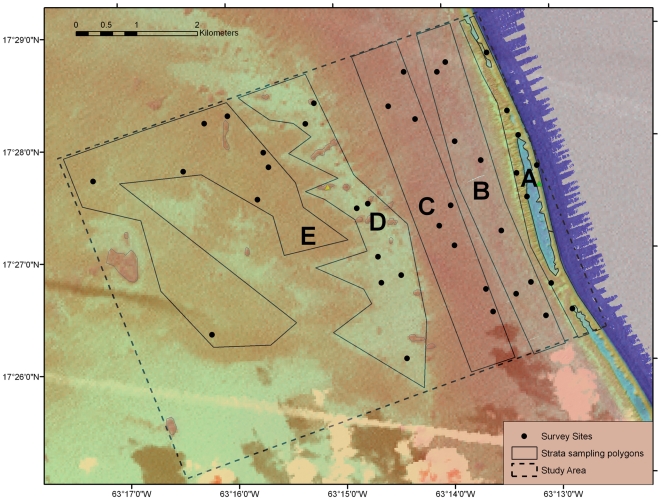
Saba Bank sampling strata and survey sites. The sampling locations are shown on a composite image of depth and ocean color. Polygons labeled A to E show the sampling strata which correspond to five habitat types: (A) fore reef, FR; (B) outer reef flat, ORF; (C) inner reef flat, IRF; (D) softbottom lagoon, LSB; (E) hardbottom lagoon, LHB. Black dots show sample locations.

The presence of each habitat type (see below) was determined using a combination of high-resolution multibeam bathymetry (Hydrographic Service of the Royal Netherlands Navy, 2006) and satellite imagery (LandSat). Spatial resolution of the bathymetric data was 2×2 m with a vertical resolution of <0.2 m. The former dataset was used to construct a bathymetric map of Saba Bank (**Figure 2**) and LandSat images were used to evaluate ocean color of shallow areas of Saba Bank. A geo-referenced LandSat image from March 26^th^ 2002 provided the best coverage of Overall Bank. Image resolution was ∼30×30 m (900 m^2^). The LandSat image was imported into GIS (ArcGIS 9.2) and superimposed on the bathymetric data layers to delineate habitat types based on reef zone, reef structure (topographic relief), and ocean color [21]. Five distinct habitat types (**Figure 3**) could be distinguished: (1) fore reef (FR); (2) outer reef flat (ORF); (3) inner reef flat (IRF); (4) softbottom lagoon (LSB) and (5) hardbottom lagoon (LHB).

At Overall Bank, a broad reef zone (>2 km in width) occurs along the bank's eastern margin. The outermost reef structure in this zone is the fore reef, with a highly variable depth profile (**Figure 1**) and a distinctive ocean color relative to adjacent reef areas (**Figure 3**). We delineated fore reef as a separate habitat type (FR) for sampling purposes. Fore reef areas deeper than 30 m were not considered. For more information on the seaward slope of Overall Bank, see Macintyre et al. [2] for geological observations and Toller et al. [22] for observations made by a remotely operated vehicle (ROV). To the east of FR, there is an expansive reef flat structure. Here, two distinct patterns of ocean coloration were observed in LandSat images: an outer (i.e. eastern) reef flat area that was light blue in color and an inner (western) reef flat area that appeared dark green. Based on these differences, we distinguished an outer reef flat habitat (ORF) from an inner reef flat habitat (IRF).

The lagoon zone (**Figure 2**) occupies much of Saba Bank's central area [3]. At Overall Bank, the lagoon floor extends west from the margin of the inner reef flat and reef slope. LandSat images showed two distinctive color patterns within the lagoon zone, i.e. light blue and dark green areas (**Figure 3**). These color differences did not overlap with identifiable bathymetric features such as patch reefs. We hypothesized that light blue color features were areas dominated by sand, whereas dark green areas represented dense algal cover on hardbottom substrate. Accordingly, we distinguished two habitat types within the lagoon zone: softbottom (LSB) and hardbottom (LHB) habitats. Note that use of the term ‘lagoon’ does not imply that central Saba Bank resembles a modern Caribbean lagoon, but simply indicates the geological origin of the atoll's central area.

Patch reefs (located inside the lagoon) and reef slope (separating inner reef flat from lagoon) were not sampled in our study due to the relatively small total area they occupy.

### Sampling Protocol: Benthic Communities

We used a random sampling design to investigate habitat types. A habitat map of the study area (see above) was used to randomly select survey positions using ArcGIS. A total of eight survey locations were selected within each of the five habitat types. An overview of all survey locations and habitat distribution is shown in **Figure 3**. In the field, survey sites were located using a WAAS-enabled GPS receiver (Garmin GPSMAP 76 or GPS 178C). Surveys were conducted from June through November 2007.

Owing to limitations of dive time and accessibility to sites on Saba Bank, our survey protocols were designed to efficiently record the habitat characteristics through a combination of quantitative and qualitative measures. Quantitative means were always used to estimate percent cover as described below. Where it was impractical to make quantitative measures, we recorded descriptive (qualitative) information that would enable us to examine for relative differences in habitat structure.

At each survey location, we examined physical characteristics and benthic community composition within a 4×25 m belt transect (100 m^2^). Percentage cover by each of the major benthic groups was estimated within each transect. The ability of each surveyor to accurately estimate benthic cover was tested beforehand using photoquadrats in which cover was measured exactly. Results from these preliminary studies indicated that estimation described 84.9% of the true variability present in reef communities. This value was deemed sufficiently high to use estimation during benthic surveys at the Saba Bank. Percentage cover of substrate (abiotic) was assigned into three categories: hardbottom (consolidated carbonate substrate), rubble (unconsolidated material of <0.5 m diameter), and sand (>0.5 cm thick layer). Percentage cover of corals, sponges, macroalgae and coralline algae was estimated to the nearest 1% and the dominant scleractinian and macroalgal genera were recorded at each sampling location. Note that abiotic and biotic descriptors were used independently to distinguish the physical composition of the substrate from that of the organisms that were observed growing on top of it.

A series of qualitative measures were also collected at each survey location in order to further characterize the relative differences in benthic composition among habitats. Vertical relief was defined as the largest vertical drop observed along the transect and it was assigned a qualitative score of low (1; <0.5 m), medium (3), or high (5; >1.0 m). Substrate rugosity was recorded into three categories of low (1), medium (3) and high (5) based on the surveyor's subjective assessment of the degree of substrate involution in relation to standardized line drawings on data forms. Depth and slope were also recorded for each location. Two qualitative descriptors were used for gorgonians: abundance (sparse, medium, dense) and height (small: 0–25 cm, medium: 25–100 cm and tall: >100 cm).

### Sampling Protocol: Fish Assemblages

Fish surveys were conducted using a belt transect visual survey protocol [23], [24]. During a 10 min survey, a diver quantified the number and size (fork length) of fishes within 2 m of the bottom in a 4×25 m belt transect. Size estimates were performed using the method of Bohnsack and Bannerot [25] and recorded in 5 cm size intervals. Length data were used to calculate fish biomass using known length-weight relationships for each species [26]. If such data did not exist, the length-weight relationship of a closely related species was used. Species were assigned to trophic guilds according to Froese and Pauly [26].

To increase the descriptive resolution of species richness of the fish assemblages at each sampling location, belt transect surveys were supplemented by roving diver (RD) surveys to provide a more complete estimate of local species richness. During RD surveys a diver swam for 10 min in a haphazardly chosen direction (i.e. approx. 100 m) and noted all fish species observed. Small-bodied demersal species (e.g. Gobiidae, Blenniidae), cryptic taxa and nocturnally active species were not included in our RD surveys because such taxa are more accurately surveyed using non-visual methods (see concurrent study by Williams et al. [17]).

### Statistical Analyses

Differences in benthic community structure were assessed using one-way ANOVA whereby the coverage of each benthic category was compared individually among the five habitat types. Differences in fish assemblage structure in terms of density, species richness and biomass were investigated using multifactor ANOVA after transformation of datasets (ln[x+1]) in order to meet assumptions of normality. Multidimensional scaling (MDS) was conducted using location-specific estimates of fish species richness and biomass to further visualize differences in fish assemblages among the five habitat types. Fish biomass estimates were transformed (ln[x+1]) and the data were standardized to equal mean (0) and standard deviation (1). The strength of the relationship between macroalgal cover and biomass of herbivorous fishes was tested by Poisson regression.

Two models of algal abundance (δ_a_) -constant, δ_a_ = α, and exponential with herbivorous biomass (H), δ_a_ = α exp(βH), where α and β are the biomass-independent and biomass-dependent terms, respectively, were compared for relative fit using a likelihood ratio test [27]. Significant overrepresentation of a species in a certain habitat type was assumed to indicate the species' preference for such habitat and was analyzed using Chi-square analyses.

## Results

### Benthic Communities

**Figure 4:**
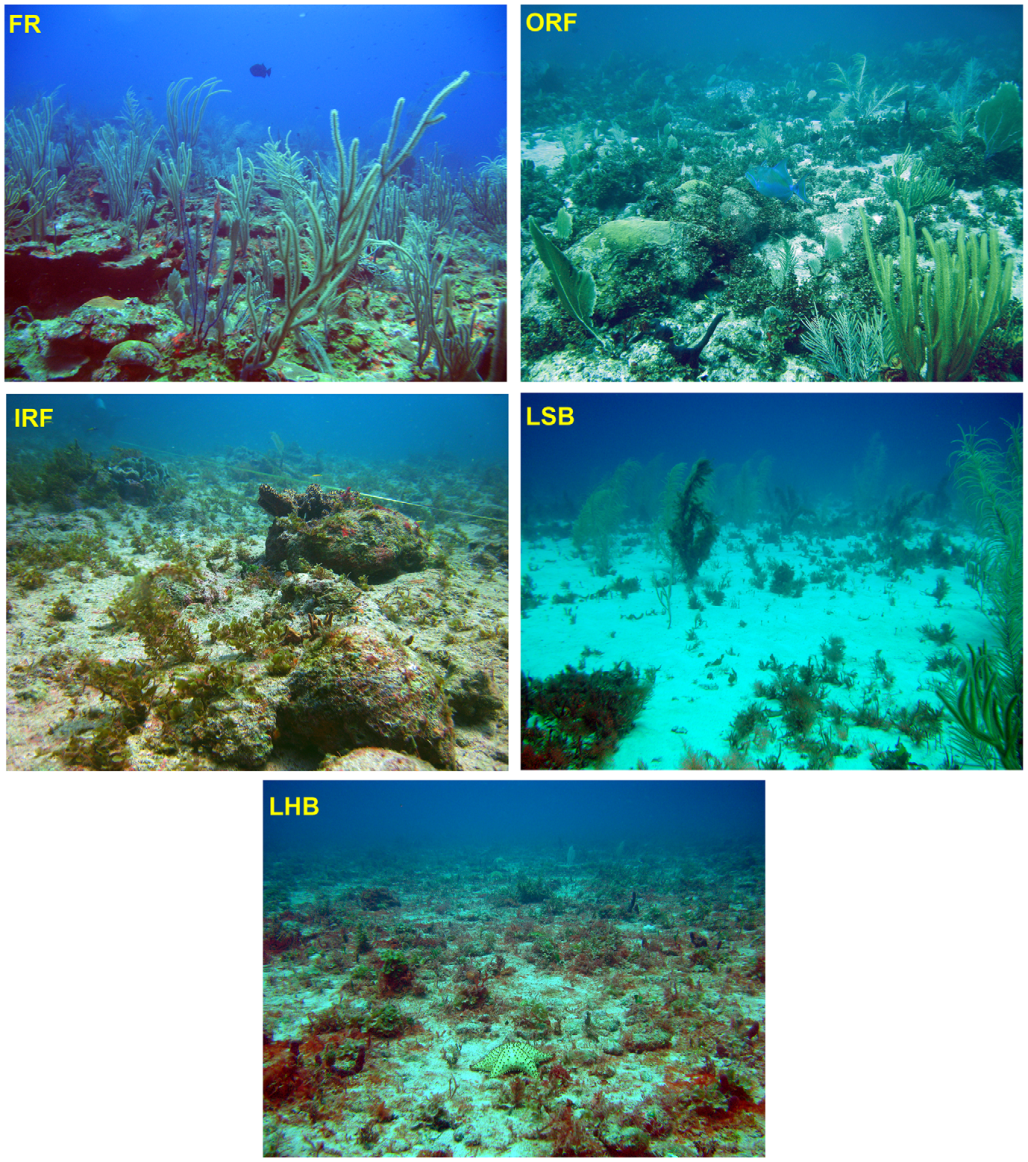
General aspect of the five habitat types found in the study area. (FR) Fore reef habitat; (ORF) outer reef flat habitat; (IRF) inner reef flat habitat; (LSB) softbottom lagoon habitat; (LHB) hardbottom lagoon habitat.

General aspects of the five Saba Bank habitat types (**Figure 4**) are summarized briefly as follows. FR habitat was comprised of complex reef structures on hardbottom substrate with high vertical relief and rugosity. Coral cover was highest in this zone. Gorgonian density was high. Macroalgae were dominated by the genera *Lobophora* and *Dictyota*. ORF habitat was comprised of hardbottom substrate or “pavements” areas with isolated structures and large rubble fragments which created some vertical relief and rugosity. Coral cover was low. Gorgonians were moderately abundant and of medium height. Dominant genera of macroalgae were *Sargassum* and *Stypopodium*. IRF habitat was comprised of low relief, hardbottom pavement areas. Rubble fragments created some vertical relief and rugosity. Coral cover was very low. Dominant genera of macroalgae were *Sargassum* and *Dictyopteris*. LSB habitat was primarily sand or sand mixed with rubble. Benthic cover was very low except for scattered gorgonians including large colonies of *Pseudopterogorgia* at some sites. The dominant macroalga was *Laurencia*. LHB habitat was comprised primarily of rubble and hardbottom, Vertical relief was low. Rubble and solution holes provided some rugosity. Corals, sponges, and gorgonians were sparse. Macroalgae were abundant and diverse with *Lobophora variegata* (ruffled form) and *Codium* dominating.

The five habitat types varied significantly in terms of physical characteristics (ANOVA; p<0.05; **Table 1**). In general, the physical structure of the bottom consisted of consolidated limestone with gradually increasing amounts of sand (especially in LSB) and rubble towards the central area of the bank (LSB, LHB). The fore reef (FR) was also structurally more complex than any of the other habitats.

**Table 1:** Physical characteristics of Saba Bank habitat types.

Category	Subcategory	Statistic	FR	ORF	IRF	LSB	LHB
Depth (m)		Avg ± StDev	23.6±3.0	13.8±0.4	13.8±0.6	19.4±0.4	20.1±0.7
		Range	20.7–29.9	13.1–14.3	12.8–14.6	18.6–20.0	19.5–21.3
Substrate (%)	Hardbottom	Avg ± StDev	87.4±14.6	56.0±42.0	68.0±29.5	2.5±7.1	44.4±37.6
		Range	55–99	0–100	5–95	0–20	0–95
		Group	a	a,b	a,b	c	b,c
	Rubble	Avg ± StDev	3.5±8.0	39.6±37.1	31.8±29.6	22.6±13.7	50.9±37.6
		Range	0–23	0–85	5–95	1–45	4–100
		Group	a	a,b	a,b	a,b	b
	Sand	Avg ± StDev	9.1±8.2	4.4±6.8	0.3±0.5	74.9±17.5	4.8±7.1
		Range	0–22	0–15	0–1	45–99	0–20
		Group	a	a	a	b	a
Vertical Relief		Avg ± StDev	4.3±1.0	1.4±0.7	1.5±0.9	1.1±0.4	1.0±0.0
		Range	3–5	1–3	1–3	1–2	1
Rugosity		Avg ± StDev	4.5±0.9	2.6±0.7	1.9±0.8	1.0±0.0	1.3±0.5
		Range	3–5	1–3	1–3	1	1–2
Slope (degrees)		Range	5–10	<1	<1	<1	<1

Abbreviations of habitat types are: (FR) fore reef; (ORF) outer reef flat; (IRF) inner reef flat; (LSB) lagoon soft-bottom; (LHB) lagoon hard-bottom. Homogenous groups are indicated with letters (a, b, c). Differences between groups were significant (p<0.05) based on one way ANOVA. Vertical relief and rugosity were assigned qualitative scores (1 to 5).

Benthic community composition also varied significantly among the five habitat types (ANOVA; p<0.05; **Table 2**). All benthic categories considered here (sponges, corals, macro- and coralline algae and gorgonians) except macroalgae and crustose coralline algae decreased in abundance from the fore reef towards the lagoonal area of the central bank (**Table 2**, **Figure 4**). The habitat types thus span a gradient along which most functional benthic groups do occur albeit in increasingly lower abundances. FR harbors the greatest diversity and abundance of benthic life forms and was likely responsible for the high topographical complexity within this habitat type (**Table 2**). Substantial cover by scleractinian corals was observed only in FR (mean coverage 11.5%), with sparse cover in ORF (2.4%) and minimal or no cover in IRF, LSB and LHB habitats (**Table 2**). Gorgonian abundance was also highest in FR, while average gorgonian height was greatest in FR and LSB (see Etnoyer et al. [28] for further information on Saba Bank gorgonians). Macroalgae were the dominant benthic group in all habitat types with mean coverage ranging between 32.5% (LSB) and 48.1% (IRF). Total cover by macroalgae did not differ significantly among habitat types, however the dominant taxa of macroalgae varied among habitat types (**Table 2**).

**Table 2:** Benthic community composition of Saba Bank habitat types.

Category	Subcategory	Statistic	FR	ORF	IRF	LSB	LHB
Benthic Cover (%)	Live Coral	Avg ± StDev	11.5±5.8	2.4±1.7	0.4±0.7	0.1±0.4	0.3±0.5
		Range	5–20	1–5	0–2	0–1	0–1
		Group	a	b	b	b	b
	Sponge	Avg ± StDev	4.2±2.2	2.0±0.5	2.1±1.7	0.4±0.5	1.8±1.5
		Range	1–8	1–3	0–5	0–1	0–5
		Group	a	b	a,b	b	b
	Macro Algae	Avg ± StDev	37.5±22.8	46.6±14.6	48.1±21.2	32.5±16.7	43.8±22.8
		Range	5–70	25–65	10–75	15–60	15–80
		Group	a	a	a	a	a
	Coralline Algae	Avg ± StDev	2.5±4.6	-	-	-	-
		Range	0–10	-	-	-	-
		Group	a	a	a	a	a
Gorgonian Assemblage	Density	Avg ± StDev	3.4±0.5	2.4±1.2	1.0±0	1.3±0.7	1.0±0
		Range	3–4	1–4	1	1–3	1
	Height	Avg ± StDev	3.1±0.4	1.9±1.0	1.3±0.7	2.9±1.7	1.0±0
		Range	3–4	1–3	1–3	1–5	1
Dominant Coral Genera			*Montastraea* (8)	*Siderastrea* (7)	none (6)	none (7)	none (7)
			*Porites* (1)	*Porites* (4)	*Dendrogyra* (1)	*Siderastrea* (1)	*Dichocoenia* (1)
			*Diploria* (1)	*Diploria* (3)	*Siderastrea* (1)		*Siderastrea* (1)
			*Siderastrea* (1)	*Montastraea* (3)			
				*Meandrina* (1)			
Dominant Algal Genera			Lobophora* (8)	*Sargassum* (8)	*Sargassum* (6)	*Laurencia* (5)	Lobophora* (5)
			*Dictyota* (6)	*Stypopodium* (3)	*Dictyopteris* (3)	*Dictyota* (1)	*Codium* (4)
			*Sargassum* (1)	*Dictyopteris* (1)	*Codium* (1)	*Sargassum* (2)	*Dictyota* (2)
							*Halimeda* (1)
							*Caulerpa* (1)
							*Dictyopteris* (1)
							*Eucheuma* (1)
							*Sargassum* (1)
							*Schizothrix* (1)

Habitat types are abbreviated as shown in Table 1. Homogenous groups are indicated with letters (a, b, c). Differences between groups were significant (p<0.05) based on one way ANOVA. Gorgonian height and density were assigned qualitative scores (1 to 5). Dominant genera of corals and macroalgae are listed with number of locations in parentheses. Two forms of *Lobophora variegata* (*) differed in distribution: a decumbent form in FR and a ruffled form in LHB.

### Fish Assemblage Structure: Belt Transects

Thirty-four commercially valued fish species [18], [19] were recorded in belt transect surveys (**Table 3**). In terms of overall abundance, the most prevalent families were Acanthuridae, Scaridae, Serranidae and Haemulidae (5.1, 3.5, 2.3 and 1.0 individuals/100 m^2^, respectively). The most frequently observed species were coney, *Cephalopholis fulva* (53% of belt transects), ocean surgeon, *Acanthurus bahianus* (50%), blue tang, *A. coeruleus* (43%), white grunt, *Haemulon plumierii* (40%), queen triggerfish, *Balistes vetula* (38%), redband parrotfish, *Sparisoma aurofrenatum* (35%) and red hind, *Epinephelus guttatus* (35%). On one FR location a large school of 200 Bermuda chubs (*Kyphosus sectator*) was observed but excluded from our analyses as an unusual observation.

**Table 3:** Comparison of fish density among Saba Bank habitat types.

Species	TG	FR	ORF	IRF	LSB	LHB
*Acanthurus bahianus*	HB	0.25±0.71 (13)	8.00±6.87 (75)	6.13±4.26 (75)	1.13±2.42 (38)	1.50±1.60 (50)
*Sparisoma aurofrenatum*	HB	2.00±2.33 (50)	4.13±4.02 (75)	2.38±2.88 (50)	-	-
*Cephalopholis fulva*	PI	1.50±1.41 (75)	4.25±3.85 (100)	1.38±1.51 (63)	0.13±0.35 (13)	0.13±0.35 (13)
*Scarus taeniopterus*	HB	2.25±2.31 (63)	3.50±3.78 (50)	-	-	-
*Acanthurus coeruleus*	HB	1.00±0.93 (63)	1.75±1.75 (75)	1.38±1.51 (50)	0.25±0.71 (13)	0.13±0.35 (13)
*Kyphosus sectator*	OM	4.38±12.37 (25)	-	-	-	-
*Haemulon plumierii*	ZB	0.88±0.99 (63)	1.00±1.41 (50)	2.00±3.66 (75)	-	0.50±1.41 (13)
*Acanthurus chirurgus*	HB	-	1.50±1.85 (50)	2.50±3.34 (63)	-	0.13±0.35 (13)
*Epinephelus guttatus*	ZB	-	1.00±0.76 (75)	1.25±1.39 (63)	0.25±0.71 (13)	0.75±1.49 (25)
*Pseudupeneus maculatus*	ZB	0.25±0.46 (25)	0.88±1.13 (50)	0.25±0.46 (25)	-	1.75±4.56 (25)
*Balistes vetula*	ZB	0.13±0.35 (13)	0.88±0.64 (75)	1.00±0.93 (63)	0.25±0.46 (25)	0.13±0.35 (13)
*Sparisoma viride*	HB	1.88±1.64 (75)	0.25±0.46 (25)	-	-	-
*Melichthys niger*	PL	1.63±2.26 (50)	-	-	-	-
*Caranx crysos*	PI	1.25±2.82 (25)	-	-	-	-
*Sphyraena barracuda*	PI	0.25±0.46 (25)	0.50±0.53 (50)	0.25±0.71 (13)	0.13±0.35 (13)	-
*Holacanthus tricolor*	ZB	0.25±0.46 (25)	0.88±1.13 (50)	-	-	-
*Holocentrus adscensionis*	ZB	-	-	0.38±1.06 (13)	-	0.50±0.93 (25)
*Caranx ruber*	PI	-	0.38±0.74 (25)	-	0.38±1.06 (13)	-
*Cephalopholis cruentata*	PI	0.63±0.74 (50)	-	-	-	-
*Ocyurus chrysurus*	PI	-	-	0.13±0.35 (13)	-	0.50±1.07 (25)
*Holocentrus rufus*	ZB	-	0.38±1.06 (13)	-	-	-
*Lutjanus mahogoni*	PI	0.38±1.06 (13)	-	-	-	-
*Sparisoma chrysopterum*	HB	-	0.38±0.74 (25)	-	-	-
*Scarus iseri*	HB	0.38±0.74 (25)	-	-	-	-
*Haemulon aurolineatum*	OM	0.38±0.74 (25)	-	-	-	-
*Haemulon melanurum*	ZB	-	0.25±0.71 (13)	-	-	-
*Mulloidichthys martinicus*	ZB	0.25±0.46 (25)	-	-	-	-
*Caranx lugubris*	PI	0.13±0.35 (13)	-	-	-	-
*Haemulon flavolineatum*	ZB	0.13±0.35 (13)	-	-	-	-
*Ginglymostoma cirratum*	ZB	-	-	0.13±0.35 (13)	-	-
*Scarus vetula*	HB	0.13±0.35 (13)	-	-	-	-
*Malacanthus plumieri*	ZB	-	-	-	0.13±0.35 (13)	-
*Lactophrys triqueter*	ZB	0.13±0.35 (13)	-	-	-	-
*Bodianus rufus*	ZB	0.13±0.35 (13)	-	-	-	-

Fish density (No. individuals/100m2) is reported as the average ± standard deviation (frequency) from 8 belt transects per habitat. Trophic Guild (TG) is: (HB) herbivore; (PI) piscivore; (PL) planktivore; (OM) omnivore; (ZB) zoobenthivore. Habitat types are abbreviated as shown in Table 1.

**Figure 5:**
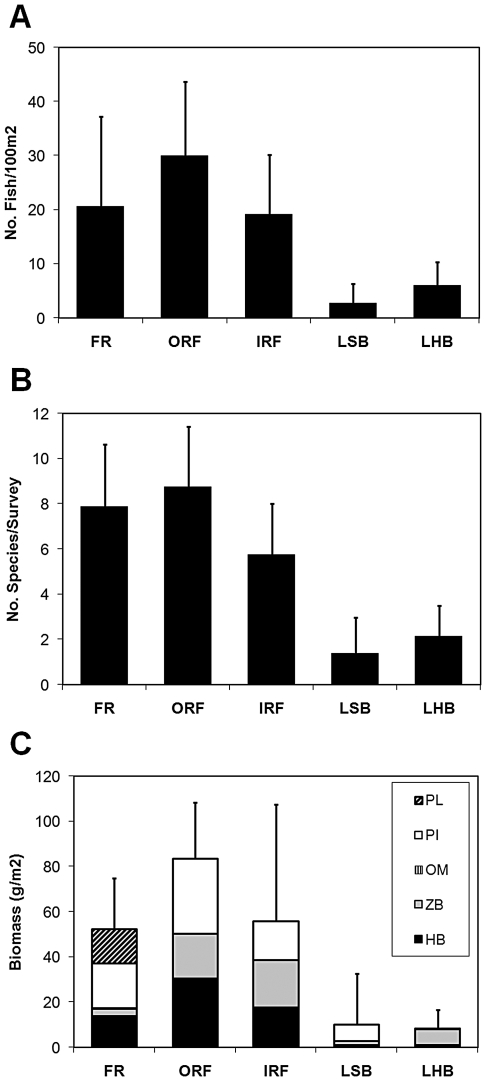
Fish assemblage structure across Saba Bank habitat types. Results of belt transect visual surveys are shown for each of the five different habitat types for: A) fish density; B) fish species richness; and C) estimates of biomass. The average values are presented for each habitat type (eight surveys per habitat). Error bars show standard deviation. Habitat types are abbreviated as in Figure 3. Trophic guilds are: planktivore (PL); piscivore (PI); omnivore (OM); zoobenthivore (ZB); herbivore (HB).

**Figure 6:**
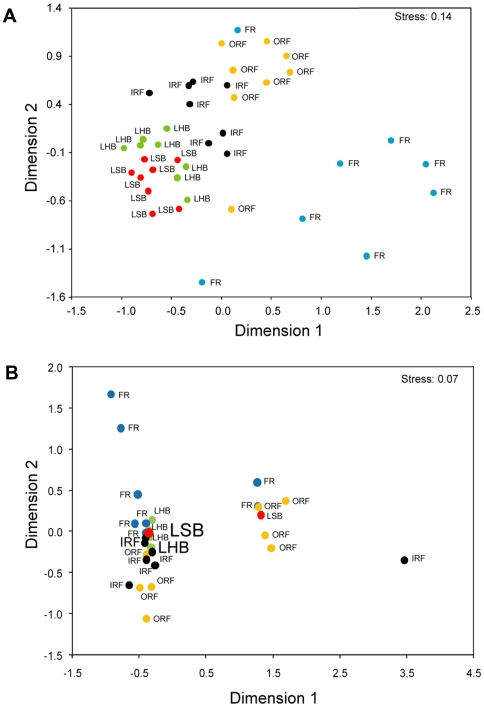
Multidimensional Scaling (MDS) analysis of fish assemblage structure. MDS plot of: A) fish species diversity; and B) fish biomass at each sampling location for each of the five habitat types. Habitat types are abbreviated as in Figure 3. Note that in B) many locations overlap. A larger number of overlapping points is indicated by an increasingly larger font size.

Fish assemblage structure was compared between habitat types based on estimates of fish density, species richness and biomass from belt transect surveys (**Figure 5**, **Figure 6**). Average fish density was highest in ORF (29.9 individuals/100 m^2^), intermediate in FR and IRF (20.5 and 19.1 individuals/100 m^2^, respectively) and lowest in the lagoonal habitat types LSB and LHB (2.6 and 6.0 individuals/100 m^2^, respectively; **Figure 5A**). Fish densities differed significantly among habitat types (F_4,35_ = 14.69, p<0.001) and post-hoc tests (Tukey) revealed that FR, ORF, and IRF harbored significantly higher densities of fish than the lagoonal habitat types LSB and LHB. We examined the potential for a single dominant species to influence trends in the dataset by excluding *A. bahianus* and repeating analyses. However results were similar, suggesting that the observed pattern does not arise from the dominant fish species.

Species richness, defined here as the average number of species observed per belt transect, was highest in FR and ORF (7.9 and 8.8 species/100 m^2^, respectively), intermediate in IRF (5.8 species/100 m^2^) and lowest in LSB and LHB (1.4 and 2.1 species/100 m^2^, respectively). Similar to fish density, species richness also differed between the five habitat types (F_4,35_ = 18.16, p<0.001; **Figure 5B**) and post-hoc tests (Tukey) revealed that FR, ORF, and IRF harbored significantly higher numbers of fish species than the lagoonal habitat types LSB and LHB. MDS analysis showed that the species composition of fish assemblages varied among habitat types: assemblages of the fore reef habitat were most distinct from all other habitat types whose fish communities became more similar towards the center of the bank (**Figure 6A**).

Fish biomass (**Figure 5C**, **Figure 6B**) also differed among habitat types (F_4,35_ = 8.22, p<0.001) whereby the habitat types FR, ORF and IRF harbored the highest fish biomass ranging between 52 to 83 grams per m^2^. Biomass estimates for lagoonal habitats were significantly lower (8 to 10 g/m^2^) compared to FR, ORF and IRF habitat types (Tukey; p<0.01). MDS analysis of fish biomass estimates also indicated that the assemblages of FR, OFR and IRF habitat types differed from those of lagoonal habitat types (**Figure 6B**).

The relative biomass of different trophic guilds also differed among habitat types (**Figure 5C**). Planktivorous fish were almost exclusively found near the edge of Saba Bank (FR), whereas zoobenthivores comprised 87% of the fish biomass in lagoonal hardbottom habitats (LHB). Biomass of herbivorous fishes correlated negatively with the abundance of macroalgae and a negative exponential model (Macroalgae (%) = 0.3786*exp(-0.0036*herbivorous fish biomass [in g/m^2^]) was strongly favored over the nested model with constant algal biomass (p<0.001). Piscivorous fishes comprised 17.4 to 33.2% of the total fish biomass in FR, ORF and IRF habitat types. Piscivore biomass was lowest in LHB (<1.2%) and predominant in LSB (>70%), however the total fish biomass in these latter two habitat types was considerably lower than in FR, ORF and IRF habitat types.

### Fish Assemblage Structure: Roving Diver Surveys

The Roving Diver (RD) survey was included to strengthen local estimates of fish species richness at a locale. A total of 97 daily active, non-cryptic fish species were recorded from our 40 RD surveys (pooled data from the five habitat types; **Table S1**). The cumulative number of species observed was greatest in FR (72 species), intermediate in ORF and IRF (54 and 46 species, respectively), and lowest in LSB and LHB (29 and 33 species, respectively). Analysis of species richness (i.e. the number of species observed per RD survey) indicated that fish assemblages differed significantly among habitat types (one-way ANOVA, F_4,35_ = 14.81, p<0.001). Average species richness was greatest in FR (28.3 spp./survey), ORF (28.8 spp./survey), and IRF (22.4 spp./survey) and lowest in LSB and LHB (10.5 spp./survey and 13.5 spp./survey, respectively). Post-hoc (Tukey) tests confirmed that species richness was significantly higher in FR, ORF and IRF habitat types than in LSB and LHB habitats.

The most commonly observed fish species were bicolor damselfish, *Stegastes partitus* (observed during 85% of all sampled locations; n = 40); ocean surgeon, *Acanthurus bahianus* (80%); queen triggerfish, *Balistes vetula* (70%); and bluehead, *Thalassoma bifasciatum* (67.5%). Three additional species, (white grunt, *Haemulon plumierii*, yellowhead wrasse, *Halichoeres garnoti*, and blackear wrasse, *H. poeyi*) were each observed in 65% of locations (**Table 4**).

**Table 4:** Comparison of fish distribution among Saba Bank habitat types: roving diver survey results for predominant species.

Species	FR	ORF	IRF	LSB	LHB	Signif.*
*Acanthurus coeruleus*	7	7	7	2	1	+
*Cephalopholis fulva*	6	8	8	1	1	+
*Sparisoma aurofrenatum*	7	8	8	-	-	+
*Holocentrus rufus*	4	7	7	-	1	+
*Holacanthus tricolor*	7	7	2	-	-	+
*Chromis cyanea*	7	5	3	-	1	+
*Serranus tigrinus*	6	5	4	-	1	+
*Scarus taeniopterus*	8	6	1	-	-	+
*Chaetodon striatus*	6	5	2	-	-	+
*Sparisoma viride*	6	4	-	-	-	+
*Serranus baldwini*	-	-	2	5	5	+
*Holocentrus adscensionis*	-	2	6	2	8	+
*Stegastes partitus*	7	7	8	5	7	ns
*Acanthurus bahianus*	4	7	8	6	7	ns
*Balistes vetula*	5	8	7	4	4	ns
*Thalassoma bifasciatum*	7	7	8	-	5	ns
*Haemulon plumierii*	6	8	8	-	4	ns
*Halichoeres garnoti*	6	7	4	3	6	ns
*Halichoeres poeyi*	1	7	7	6	5	ns
*Malacanthus plumieri*	1	7	6	8	3	ns
*Epinephelus guttatus*	2	8	8	1	5	ns
*Halichoeres bivittatus*	1	5	5	7	5	ns
*Sphyraena barracuda*	6	5	4	2	4	ns
*Pseudupeneus maculatus*	4	6	3	1	4	ns
*Sparisoma radians*	-	5	3	4	5	ns
*Acanthurus chirurgus*	-	7	5	2	2	ns
*Halichoeres maculipinna*	1	7	5	2	1	ns
*Sparisoma atomarium*	1	5	2	2	6	ns

Habitat types are abbreviated as shown in Table 1. (*) Results of Chi square test for significance of observed distributions among habitat types; (+)  =  significant at p>0.05; (ns)  =  not significant. Results shown here include the 28 most commonly observed species from a total of 97 species that were recorded in roving diver surveys (see **Table S1**).

Twenty-eight fish species were sufficiently represented in RD surveys to investigate potential habitat associations. Of these, 12 species were unequally distributed among habitats (Chi-square test; p<0.01) and16 species showed no significant difference (**Table 4**). Ten species were more common in habitats of the reef zone (FR, ORF and/or IRF) and uncommon or absent from the lagoon (LSB and LHB). However, two species showed an opposite pattern of distribution: the squirrelfish (*Holocentrus adscensionis*) was absent from FR, while the lantern bass (*Serranus baldwini*) was more common in LSB and LHB.

Habitat associations were also examined at the level of fish family after pooling RD survey data from all sites. The ten most common fish families were in declining order: Labridae, Scaridae, Serranidae, Acanthuridae, Pomacentridae, Holocentridae, Balistidae, Haemulidae, Chaetodontidae, and Carangidae. For eight families (**Table S1**), the hypothesis of equal distribution among habitats was tested and rejected (Chi-square test; p<0.01). Familial representation was generally highest in habitats of the reef zone (FR, ORF and/or IRF) and lowest in habitats of the lagoon zone (LSB and LHB). Serranids, pomacentrids, chaetodontids and carangids were more common in FR habitat whereas labrids and scarids were most common in ORF habitat (**Table S1**). Acanthurids and balistids showed no clear habitat associations at the family level.

## Discussion

### Benthic Communities

In this study, substantial coral cover was observed only in the fore reef (FR) zone of Overall Bank and rapidly diminished with increasing distance from the shelf edge. This suggests that significant reef accretion is restricted to a narrow zone along the Bank's periphery. Van der Land [3] also found that corals from the lagoon zone of Saba Bank were “small in size and number,” but concluded that substrate was not a limiting factor for coral settlement. It is probable that coral populations experience high post-settlement mortality due to normal heavy wave conditions and sand scour associated with storm events. As such, the central lagoon of Saba Bank would represent a natural “marginal” habitat for coral growth (*sensu* Vermeij et al. [29]). Meesters et al. [1] examined coral cover on the fore reef slope of eastern Saba Bank, including three sites at Overall Bank, and reported coral cover of 60 to 90%. Klomp and Kooistra [4] also examined benthic communities from three sites at Overall Bank and reported 11, 26, and 41% coral cover. In the present study, we observed average coral cover in fore reef habitat that was appreciably lower (11.5%) than previously reported. Anecdotal observations suggest that coral cover on Saba Bank has declined during the past five years. In many of our FR surveys, large recently dead (or partially dead) colonies of *Montastraea faveolata* and *Colpophyllia natans* were observed. We speculate that the reduced coral cover recorded on Saba Bank in 2007 may be a consequence of declines following the 2005 coral bleaching event that severely impacted reefs in the northern Caribbean [30].

Other reports already noted the unusual diversity and abundance of macroalgae on Saba Bank [31] and our surveys confirm that macroalgae are the most dominant component in benthic communities of all surveyed habitats. While herbivorous fish exert some controlling effect on local abundance of macroalgae as suggested by the negative correlation between herbivorous fish biomass and algal abundance, it remains unclear what environmental conditions are responsible for the abundance of various species of macroalgae on Saba Bank. Macroalgal domination and low coral cover are common features of reefs exposed to high wave energy elsewhere in the region [32], [33]. Predominance of macroalgae on Saba Bank might therefore be expected due to the bank's unprotected position in the open ocean. However, despite the apparently favorable growth conditions for marine macrophytes on Saba Bank, no seagrasses were observed at any survey locations in this or other studies of Saba Bank [1], [3], [34]. Thus, there is no indication from available data that seagrass beds form a significant component of the Saba Bank marine ecosystem, which likely has consequences for fish species that depend on these marine habitats as “nursery areas” [11].

### Fish Assemblage Structure

Fish assemblages from the five Saba Bank habitat types differed in terms of species richness, density, biomass, and trophic structure (**Figure 5**, **Figure 6**). Elsewhere, studies have shown that reef fish abundance and diversity are strongly influenced by habitat complexity [16], [35]–[37]. Indeed, this hypothesis is generally supported by our results across a gradient of Saba Bank habitats. We found that reef fish abundance and diversity were high in complex habitats of the reef zone (FR, ORF, and IRF) when compared to lagoon (LSB, LHB) where vertical relief was minimal and corals were absent. This suggests that Saba Bank's coral reefs play an important role in supporting reef fishes including commercially valued species.

Fish assemblages observed at the Saba Bank reef margin were exceptional in that they did not corroborate the hypothesized relation of habitat complexity to fish abundance and diversity. The fore reef showed the greatest habitat complexity (coral cover, vertical relief and rugosity) yet fish diversity and biomass were similar to or lower than in the adjacent reef flat habitat. The reason for this remains unclear. Commercial trap fishing may have acted selectively to reduce fish density and biomass in the bank's fore reef areas. Alternatively, natural productivity rates on the reef flat may be greater than on the fore reef.

Newman et al. [38] recently studied fish biomass in a range of coral reef habitats in the Caribbean and found average biomass estimates to range between 15 and 60 g/m^2^. Biomass estimates for Saba Bank ranged between 52 and 83 g/m^2^ for habitats of the reef zone (FR, ORF and IRF) indicating that these habitat types on the Saba Bank harbor higher fish biomass than most locations in the Caribbean region. Sandin et al. [15] found that within the Caribbean basin, species richness of island fish faunas fits the classical species-area relationship and is governed by island biogeographic factors such as remoteness from source populations and the available number of different habitat types. The remoteness of Saba Bank could hence explain the lower than expected number of fish species present (i.e. 115 species relative to Caribbean islands of similar size that have either nursery habitats or are more closely located to larger land masses [15]). It needs to be noted fish species not considered in our study (i.e. cryptic and nocturnal species) were excluded from the Sandin et al. [15] dataset before we compared the two datasets.

Many typical coral reef fish species are highly dependent on nursery habitats such as mangroves and sea grass beds [12], [39], [40]. Coral reef islands that lack mangroves and seagrass beds have been found to have markedly lower species richness of those taxa (such as many lutjanids, haemulids and scarids) that depend most strongly on such habitats [41]. The same appears to be true for Saba Bank where the rarity (sea grass beds) or absence (mangroves) of important nursery habitats likely explains the relative scarcity of certain species such as yellowtail snapper, *Ocyurus chrysurus*, schoolmaster, *Lutjanus apodus*, rainbow parrotfish, *Scarus guacamaia*, and striped parrotfish, *S. iseri*, or the absence of species such as gray snapper, *L. griseus*, bluestripe grunt, *Haemulon sciurus*, and yellowfin mojarra, *Gerres cinereus*. Two grunt species (white grunt, *H. plumierii*, and cottonwick, *H. melanurum*) were abundant in our surveys but overall haemulid diversity on Saba Bank was low. Species richness of other common Caribbean reef fish families was also relatively low on Saba Bank (Gerreidae, none observed; Sparidae,1 species; Lutjanidae, 3 species) - many of which require either seagrasses and/or mangroves as “nursery areas” [11], [41], [42]. Fisheries-dependent observations further confirm the rarity of such species [18], [19]. Thus the absence of important nursery habitats appears to be reflected in reduced species richness of Saba Bank fish assemblages.

Large piscivores and apex predators were abundant compared to elsewhere in the region [38]. In the 40 RD surveys on Saba Bank, we recorded 63 mid- to large sized groupers, carangids, barracudas and/or (nurse) sharks. The abundance of some predatory fishes stems from the fact that they are not targeted by the commercial fishery. For example, the great barracuda, *Sphyraena barracuda*, is a large piscivore that is generally not harvested by commercial fishermen because of concerns about ciguatera poisoning. Barracuda were common in visual surveys (>50% of surveys) and observed in all habitat types. Notwithstanding reliable yet anecdotal historical observations, which suggest that fishing has negatively impacted several large piscivore populations on the bank [18], the observed abundance of large predatory fish can be considered as an indicator of the relatively good ecosystem health of Saba Bank, relative to most reefs in the Caribbean region at present [42]–[45].

Our observations from Saba Bank indicate a simultaneous abundance of predatory fishes and macroalgae in the same area, which is remarkable. Common trajectories of Caribbean reef decline require the removal of the highest trophic levels (e.g. predatory fishes) so biomass production at lower trophic levels (e.g. macroalgae) is no longer transformed through various trophic linkages to large and long-lived organisms such as corals and large predatory fish [45]. Saba Bank does not seem to fit this paradigm making it an interesting location to study trophic linkages in Caribbean reef systems.

### Conclusions

Results of this study provide an initial description of typical fish assemblages from one area of Saba Bank, and give some indication of the role that habitat plays in structuring the composition and abundance of assemblages. Keeping in mind that the Overall Bank study area (40 km^2^) represents only 1.8% of Saba Bank's total area, generalizations must be made with caution. Further exploration is required to quantify the distribution of natural resources on Saba Bank and to elucidate the ecological processes which are at play in the biological communities of this unique ecosystem. Detailed benthic habitat maps have already been developed for some Caribbean reef ecosystems [21], [46]–[47]. Our results will provide support for similar efforts to map habitats of Saba Bank in the future [20].

Findings presented here raise questions about the importance of nursery habitats for some fish species and the role of disturbance in structuring benthic communities. From the perspective of managing fisheries resources, it is essential to understand which habitat types serve as alternative nursery areas for commercially valued species, and whether such areas require special protective measures. Factors that have contributed to the good ecosystem health of Saba Bank likely include the Bank's inaccessibility, its distance from major coastal sources of pollution, the small size of the fishing fleet operating on the bank, and the large size of the bank itself. However, formal protection and strategic management of Saba Bank will ensure that anthropogenic stressors do not lead to degradation of this unique ecosystem in the future.

## Supporting Information

Table S1 Fish species number across Saba Bank habitat types* as observed in roving diver surveys. Values are the number of location within each habitat type where a species was observed (8 surveys per habitat type). (*) Abbreviations for habitat types are: FR  =  fore reef; ORF  =  outer reef flat; IRF  =  inner reef flat; LSB  =  lagoon soft-bottom; LHB  =  lagoon hard-bottom. (**) Chi square test to determine the statistical significance of observed species distribution among habitat types. NS  =  not significant; (+)  =  significant at p>0.05; (++)  =  significant at p>0.01. The dagger symbol indicates a significant difference in family-level distribution among habitat types. For Acanthuridae and Balistidae, differences observed in among-habitat distribution were not significant (ns).
(0.03 MB XLS)
